# Cholesterol Alters the Phase Separation in Model Membranes Containing hBest1

**DOI:** 10.3390/molecules27134267

**Published:** 2022-07-02

**Authors:** Pavel Videv, Kirilka Mladenova, Tonya D. Andreeva, Jong Hun Park, Veselina Moskova-Doumanova, Svetla D. Petrova, Jordan A. Doumanov

**Affiliations:** 1 Faculty of Biology, Sofia University “St. Kliment Ohridski”, 8 Dragan Tzankov Str., 1164 Sofia, Bulgaria; pvidev@uni-sofia.bg (P.V.); k_mladenova@biofac.uni-sofia.bg (K.M.); phun@uni-sofia.bg (J.H.P.); moskova@biofac.uni-sofia.bg (V.M.-D.); spetrova@biofac.uni-sofia.bg (S.D.P.); 2 Institute of Biophysics and Biomedical Engineering, Bulgarian Academy of Sciences, Acad. G. Bonchev Str., Bl. 21, 1113 Sofia, Bulgaria; t_andreeva@abv.bg; 3 Faculty of Applied Chemistry, Reutlingen University, Alteburgstraße 150, 72762 Reutlingen, Germany

**Keywords:** hBest1, cholesterol, POPC, SM, phase separation

## Abstract

Human retinal pigment epithelial (RPE) cells express the transmembrane Ca^2+^-dependent Cl^−^ channel bestrophin-1 (hBest1) of the plasma membrane. Mutations in the hBest1 protein are associated with the development of distinct pathological conditions known as bestrophinopathies. The interactions between hBest1 and plasma membrane lipids (cholesterol (Chol), 1-palmitoyl-2-oleoyl-sn-glycero-3-phosphocholine (POPC) and sphingomyelin (SM)) determine its lateral organization and surface dynamics, i.e., their miscibility or phase separation. Using the surface pressure/mean molecular area (π/A) isotherms, hysteresis and compressibility moduli (C_s_^−1^) of hBest1/POPC/Chol and hBest1/SM/Chol composite Langmuir monolayers, we established that the films are in an LE (liquid-expanded) or LE-LC (liquid-condensed) state, the components are well-mixed and the Ca^2+^ ions have a condensing effect on the surface molecular organization. Cholesterol causes a decrease in the elasticity of both films and a decrease in the ΔG_mix_^π^ values (reduction of phase separation) of hBest1/POPC/Chol films. For the hBest1/SM/Chol monolayers, the negative values of ΔG_mix_^π^ are retained and equalized with the values of ΔG_mix_^π^ in the hBest1/POPC/Chol films. Shifts in phase separation/miscibility by cholesterol can lead to changes in the structure and localization of hBest1 in the lipid rafts and its channel functions.

## 1. Introduction

Retinal pigment epithelial (RPE) cells express the transmembrane Ca^2+^-dependent Cl^−^ channel human bestrophin-1 (hBest1) on the basolateral domains of the plasma membrane [1,2,3,4]. In the central nervous system, hBest1 is involved in the permeability of glutamate and γ-aminobutyrate [4,5]. Mutations in the hBest1 protein are associated with the development of numerous pathologies such as bestrophinopathies, Alzheimer’s, Parkinson’s and other diseases [1,2,3,4,5,6,7,8,9,10,11].

The lateral membrane heterogeneity in the eukaryotic cells is a consequence of different interaction activities between lipids and/or proteins in plasma membranes [12,13]. The coexistence of different phase-separated domains (phase separation): liquid-ordered (L_o_, which also include “lipid and/or membrane rafts”) and liquid-disordered (L_d_), determines heterogeneity, structure and membrane functions [14,15,16,17,18]. 

Phospholipids, including sphingolipids, are the main lipids of plasma membranes in cells and their interactions, with hBest1 determine its lateral organization and conformational dynamics [19,20,21]. The concept of “phase separation” raises the question of the role of cholesterol in the miscibility and/or formation of different domains containing hBest1 and lipids, such as POPC or SM, in surface monolayers. 

Recently, we have demonstrated the condensing effect of cholesterol (Chol) on hBest1/POPC (1-palmitoyl-2-oleoyl-sn-glycero-3-phosphocholine) and hBest1/SM (sphingomyelin) composite monolayers as well as the crucial role of Ca^2+^ ions in this process [22].

Here, we describe the surface dynamics of ternary hBest1/POPC/Chol and hBest1/SM/Chol Langmuir monolayers using the surface pressure/mean molecular area (π/A) isotherms, hysteresis and compressibility moduli (C_s_^−1^). The integration of π/A isotherms allows us to determine the total free energy of mixing the ΔG_mix_^π^ values (the thermodynamic preferences for miscibility or separation) of the three types of molecules: hBest1, POPC, or SM, in the presence of Chol in order to throw a light on structure–function relationship.

## 2. Results and Discussion:

### 2.1. Surface Properties of Binary Monolayers Containing Cholesterol

**Figure 1:**
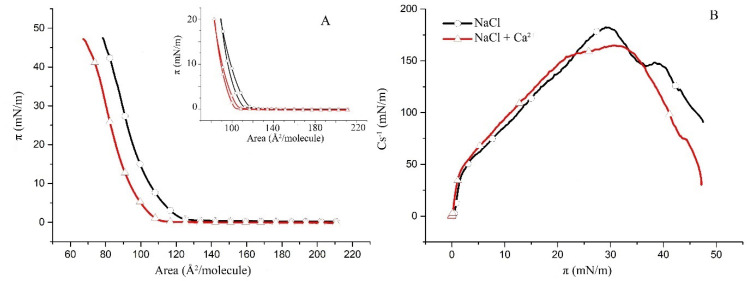
(**A**) Surface pressure/mean molecular area isotherms of the binary POPC/Chol (1:1) monolayers in a subphase of 150 mM NaCl (black) and in 150 mM NaCl supplemented with 0.5 µM CaCl_2_ (red) at 35 ± 2 °C (inset: π/A hysteresis cycles of compression–decompression); (**B**) surface compressibility moduli C_s_^−1^ derived from the isotherms in (**A**) as a function of surface pressure.

**Figure 2:**
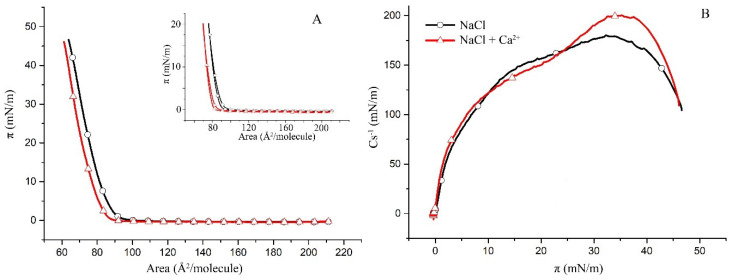
(**A**) Surface pressure/mean molecular area isotherms of the binary SM/Chol (1:1) monolayers in a subphase of 150 mM NaCl (black) and in 150 mM NaCl supplemented with 0.5 µM CaCl_2_ (red) at 35 ± 2 °C (inset: π/A hysteresis cycles of compression–decompression); (**B**) surface compressibility moduli C_s_^−1^ derived from the isotherms in (**A**) as a function of surface pressure.

**Figure 3:**
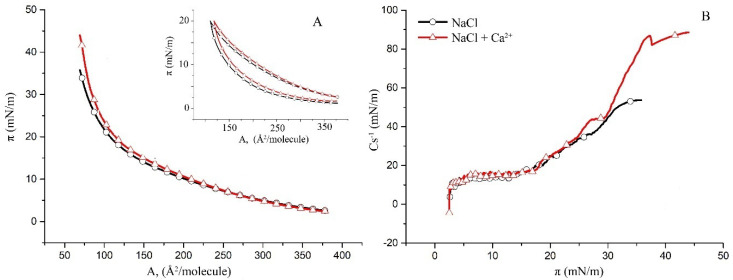
(**A**) Surface pressure/mean molecular area isotherms of the binary hBest1/Chol (1:58.5) monolayers in a subphase of 150 mM NaCl (black) and in 150 mM NaCl supplemented with 0.5 µM CaCl_2_ (red) at 35 ± 2 °C (inset: π/A hysteresis cycles of compression–decompression); (**B**) surface compressibility moduli C_s_^−1^ derived from the isotherms in (**A**) as a function of surface pressure.

This study follows a bottom-up approach by first examining the less complex binary subsystems of POPC/Chol, SM/Chol and hBest1/Chol as a basis for the more complex ternary hBest1/POPC/Chol and hBest1/SM/Chol systems. The surface pressure versus molecular area (π/A) isotherms of the binary monolayers (presented in Figure 1A, Figure 2A and Figure 3A) show a gradual increase in the surface pressure and molecular packing accompanying the reduction in mean molecular area during compression and without indications for phase transitions such as plateaus or kinks. However, the three isotherms have different shapes and courses.

The compression π/A isotherms of the POPC/Chol and SM/Chol monolayers with addition of Ca^2+^ are shifted towards smaller molecular areas at a given π compared to the isotherms without Ca^2+^. The shape and slope of both the POPC/Chol and SM/Chol isotherms are not changed; therefore, the presence of Ca^2+^ does not affect the phase state and molecular organization in these two binary monolayers. Other features of interest in the π/A isotherms are the collapse surface pressure (π_col_) and the collapse area per molecule (A_col_). While π_col_ is not affected by the addition of Ca^2+^ ions, A_col_, which is a measure of the condensing effect of the Ca^2+^ ions, is decreased by 10 Å^2^/molecule in the POPC/Chol monolayer (Figure 1A) and by only 4 Å^2^/molecule in the SM/Chol monolayer. 

The condensing effect of Ca^2+^ ions is also reflected in the reduction of the hysteresis of the binary POPC/Chol (Figure 1A, inset) and SM/Chol (Figure 2A, inset) monolayers, as again, this effect was stronger in the first system. 

The monolayer hysteresis results from the interplay between the hydrophilic–lipophilic balance of the lipid molecules, molecular cohesion and polar head group–subphase interaction determine the molecular packing and viscoelastic properties of the monolayer at the interface during compression and decompression. In essence, the binding of Ca^2+^ affects each of these parameters and the balance between them in the direction of compaction and increases the reversibility of the molecular reorganizations occurring during the compression and decompression of the monolayers.

The condensing effect of the Ca^2+^ ions on the SM monolayers [23,24] and on the Chol monolayers [25] is already well evidenced. By a combination of experimental (π/A isotherms) and theoretical (molecular dynamics simulations) studies, it was shown that the addition of Ca^2+^ does not change the shape of the π/A isotherm of Chol but displaces it to a considerably lower mean molecular area. 

The zeta potential in the POPC vesicles was found to increase from 0 mV in pure water to 15 mV after the addition of CaCl_2_, thus indicating the adsorption of Ca^2^+ ions [26]. However, the effect was found to depend on the concentration of Ca^2+^ ions, thus giving rise to a 5% reduction in the mean area per one POPC molecule at a concentration of 0.1 M Ca^2+^. There was no noticeable effect of Ca^2+^ on the POPC monolayers at a Ca^2+^ concentration of 0.5 µM that we could observe [20]; hence, the condensing effect of Ca^2+^ on the binary POPC/Chol monolayers mainly originated from the effect of the Chol molecules. 

Both the maxima of the compressional moduli C_s_^−1^ in the POPC/Chol monolayers without and with Ca ions in the subphase (Figure 1B) fall within the limits (from 100 to 250 mN/m) defined for a liquid-condensed phase [27]. C_s_^−1^ is a measure for the compressional elasticity of the layer, which is practically unaffected by the addition of Ca^2+^; the maximal C_s_^−1^ is reduced by only 15 mN/m and shifted to a slightly higher surface pressure. 

The maximal compressional moduli of the SM/Chol monolayers, presented in Figure 2B, indicate that the monolayers are in the same liquid-condensed (LC) phase. Maximal C_s_^−1^ is, in this case, increased by about 20 mN/m and also shifted to a slightly higher surface pressure. The shift to a higher π implies a slight, Ca-promoted stabilization of these binary monolayers. A characteristic feature of an SM monolayer is the liquid-expanded (LE)-LC phase transition, which is manifested by a broad plateau (at surface pressures from 6 to 15 mN/m) in the C_s_^−1^/π plot [28]. Such a plateau is absent in the SM/Chol monolayers, thus suggesting the molecular mixing of both lipids.

The maximal compressibility modulus of the SM monolayers at 35 °C is about 50 mN/m [29] (both without and with Ca^2+^ in the subphase), and an addition of Chol in 1:1 molar ratio causes 3.5, or fourfold increase of this value correspondingly, thus pushing the phase state from LE to LC. 

Two different regions are distinguished in the π/A isotherms of the hBest1/Chol (molar ratio of 1:58.5, corresponding to a surface area ratio of 1:3) monolayers in Figure 3A. At low surface pressures up to around 20 mN/m, the isotherm resembles that of pure hBest1 [20,29], while at higher surface pressures, it appears similar to the isotherm of Chol. Both isotherms have identical shapes and an equilibrium-spreading surface pressure of π_0_ = 2.6 mN/m and match with each other up to π~13 mN/m. Above 13 mN/m, the hBest1/Chol isotherm with added Ca^2+^ shifts to slightly higher molecular areas in contrast to the POPC/Chol and SM/Chol monolayers, which shift to lower areas. 

Cholesterol monolayers on different water and buffer subphases and temperature conditions have been studied in various works [30,31,32]. The maximum compression modulus of the Chol monolayer (C_s_^−1^(max)) reached at π 35 mN/m was found to be 53 mN/m upon the addition of hBest1 (Figure 3B), which indicates that the monolayers become more disordered. However, the addition of Ca ions improves the molecular order in the hBest1/Chol monolayer, as evidenced by the increased C_s_^−1^(max)~90 mN/m. Contrariwise, the maximum C_s_^−1^ of the hBest1/Chol monolayers without and with Ca^2+^ is four and eight times higher, respectively, compared to the pure hBest1 films [29], suggesting a strong reduction in the elasticity and fluidity of the protein film that is related to the condensing role of Chol and Chol+Ca^2+^.

π/A compression–decompression cycles of the hBest1/Chol monolayers, presented in the inset of Figure 3A, confirm the aforementioned findings that the addition of hBest1 decreases the ordering and increases the fluidity of the Chol monolayer. The Chol monolayer shows almost zero hysteresis [33], but when mixed with hBest1, the hysteresis becomes significant and similar to that of the hBest1 and hBest1/POPC monolayers [20,29]. The shape of the compression–decompression cycles and the magnitude of the hysteresis are not affected by the presence of Ca^2+^. 

### 2.2. Surface Properties of hBest1/POPC/Chol and hBest1/SM/Chol Monolayers

**Figure 4:**
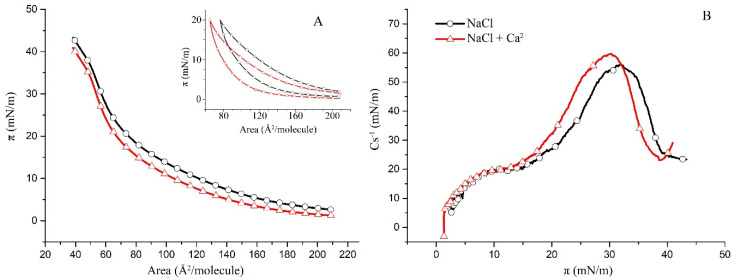
(**A**) Surface pressure/mean molecular area isotherms of the ternary hBest1/POPC/Chol (1:45:45) monolayers in a subphase of 150 mM NaCl (black) and in 150 mM NaCl supplemented with 0.5 µM CaCl_2_ (red) at 35 ± 2 °C (inset: π/A hysteresis cycles of compression–decompression); (**B**) surface compressibility moduli C_s_^−1^ derived from the isotherms in (**A**) as a function of surface pressure.

Using binary hBest1/POPC and hBest1/SM films [20,29], we built monolayers with an area ratio of hBest1/lipid of 1:3 (the monolayer area occupied by the protein to the area occupied by the lipids) in order to investigate the thermodynamic behavior of the three-component systems hBest1/POPC/Chol and hBest1/SM/Chol in biologically relevant conditions. The area ratio of 1:3 was achieved at the molar ratios of the ternary hBest1/POPC/Chol and hBest1/SM/Chol monolayers of 1:45:45 and 1:86:86, respectively, by maintaining an equimolar ratio between the two lipids as in the monolayers described in Section 2.1. The addition of hBest1 protein molecules thoroughly changes the π/A isotherm of the binary lipid POPC/Chol monolayer. In fact, its shape more resembles that of a hBest1/Chol monolayer. The π/A isotherms of hBest1/POPC/Chol monolayers have identical shapes, as “stretched” sinusoidal curves in the absence and presence of Ca^2+^ (Figure 4A). The addition of Ca ions reduces the equilibrium spreading π_0_ from 2.6 mN/m to 1.3 mN/m and shifts the whole isotherm to lower surface pressures, which indicates a condensing effect of Ca^2+^ on the surface molecular organization. A change in the course of the isotherm is observed at about 15 mN/m (π_tr_). The precise location of π_tr_ can be found on the C_s_^−1^/π curves in Figure 4B. They show a gradual increase in the compressibility modulus at low surface pressures below 13 mN/m (π_tr_) followed by a much steeper increase in C_s_^−1^ at surface pressures from 13 mN/m to 32 mN/m (for the subphase without Ca^2+^) or 30 mN/m (for the subphase with Ca^2+^), where the maximum values of C_s_^−1^ are reached. C_s_^−1^(max) was 56 mN/m for the monolayer in a NaCl subphase versus 60 mN/m for the monolayer in a NaCl subphase supplemented with CaCl_2_, which is slightly above the upper limit of 50 mN/m suggested by Davies and Rideal [27] for the LE phase. These values are higher than the C_s_^−1^(max) of the hBest1 monolayers (10.7 mN/m according to [17]) and considerably lower than the C_s_^−1^(max) of the POPC (~110 mN/m according to [34]) and the Chol (839 mN/m according to [35]) monolayers, which is an indication of mixing the three components of this ternary monolayer. The LE state of the hBest1/POPC/Chol monolayer and the reduced value of π_col_ also indicate very good mixing of the monolayer components.

**Figure 5:**
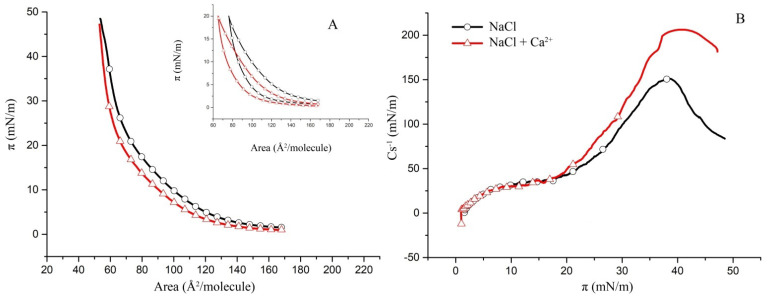
(**A**) Surface pressure/mean molecular area isotherms of the binary hBest1/SM/Chol (1:86:86) monolayers in a subphase of 150 mM NaCl (black) and in 150 mM NaCl supplemented with 0.5 µM CaCl_2_ (red) at 35 ± 2 °C (inset: π/A hysteresis cycles of compression–decompression); (**B**) surface compressibility moduli C_s_^−1^ derived from the isotherms in (**A**) as a function of surface pressure.

The isotherm of the hBest1/SM/Chol monolayer is steeper than that of the hBest1/POPC/Chol monolayer; therefore, the former is in a more condensed state. This is also reflected in the surface elasticity, which is expressed as a compressibility modulus in Figure 5B. The C_s_^−1^(max) for the hBest1/SM/Chol monolayer without Ca ions is 152 mN/m and 206 mN/m with Ca ions, which is 2.7 and 3.5 times higher than the value of the hBest1/POPC/Chol monolayer and corresponds to the LC phase state. The Ca^2+^ ions exert a considerable condensing and stabilizing effect on the hBest1/SM/Chol monolayer, as evidenced by the decrease in the monolayer elasticity.

Cholesterol is known to form highly condensed monolayers in a solid (S) phase state, with π_col_~45 mN/m [35]. Neither of the ternary monolayers studied here show the existence of an S state; therefore, the molecules of Chol are well mixed with the other components of the monolayers. 

At 35 °C and in both cases (without and with Ca ions), the molecules of hBest1 self-organize in an expanded monolayer at the air/water interfaces along the whole compression course without reaching a collapse [29]. The POPC monolayer is in the LE state up to the collapse at 46 mN/m [20]. 

The maximal values of C_s_^−1^ found for the hBest1/SM/Chol (1:86:86) monolayers are considerably higher than the C_s_^−1^(max) of the hBest1 monolayers (10.7 mN/m according to [17]) and lower than the C_s_^−1^(max) of the SM (~220 mN/m according to [36]) and the Chol (839 mN/m according to [35]) monolayers, which is an indication that the three components of this ternary monolayer have been mixed. The SM (16:0) used in this study experienced an LE-LC phase transition at surface pressure π_tr_ = 46.8 mN/m in both the NaCl and NaCl + Ca subphases [29], which disappeared when combined with hBest1 and Chol, thus supporting the claim that the components are mixed.

### 2.3. Analysis of Mixing and Phase Separation in hBest1/POPC/Chol and hBest1/SM/Chol Monolayers

**Figure 6:**
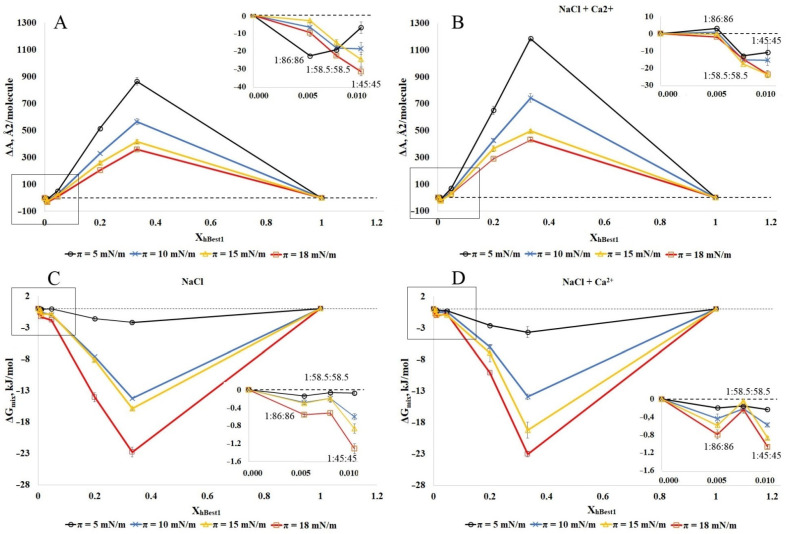
Plots of the deviation of the area from the additivity (ΔA) (**A**,**B**) and the total free energy of mixing ($∆G_{mix}^{\pi }$) (**C**,**D**) versus film composition (X_hBest1_) at different surface pressures of hBest1/POPC/Chol monolayer in a subphase of 150 mM NaCl (**A**,**C**) and in 150 mM NaCl supplemented with 0.5 µM CaCl_2_ (**B**,**D**) at 35 ± 2 °C (inset: enlargement of the framed area).

**Figure 7:**
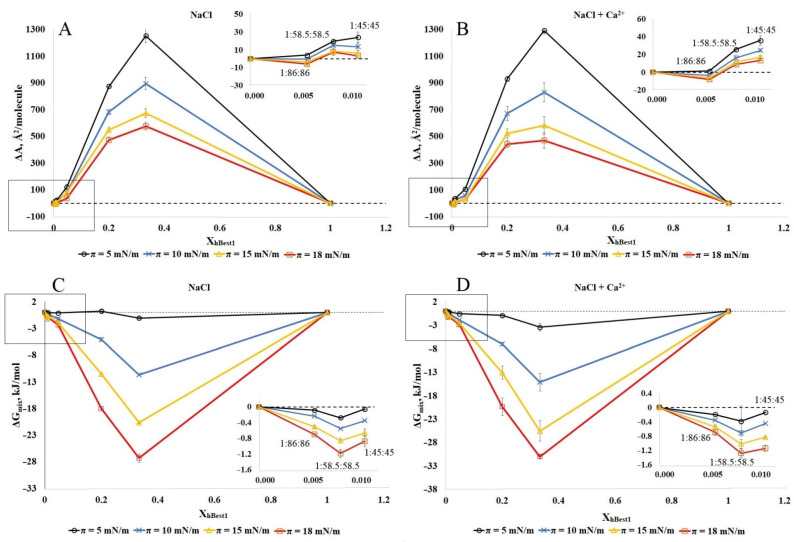
Plots of the deviation of the area from the additivity (ΔA) (**A**,**B**) and the total free energy of mixing ($∆G_{mix}^{\pi }$) (**C**,**D**) versus film composition (X_hBest1_) at different surface pressures of hBest1/SM/Chol monolayer in a subphase of 150 mM NaCl (**A**,**C**) and in 150 mM NaCl supplemented with 0.5 µM CaCl_2_ (**B**,**D**) at 35 ± 2 °C (inset: enlargement of the framed area).

To proceed with the thermodynamic analysis of the degree of miscibility between hBest1 and the lipids (POPC, SM and Chol) in the ternary monolayers, we built isotherms with various molar ratios of hBest1/lipids, as follows: 1:86:86 (X_hBest1_ = 0.006); 1:58.5:58.5 (X_hBest1_ = 0.0085); 1:45:45 (X_hBest1_ = 0.011); 1:10:10 (X_hBest1_ = 0.048); 1:2:2 (X_hBest1_ = 0.20); 1:1:1 (X_hBest1_ = 0.33), maintaining the equimolar ratio between the two lipids as in the isotherms presented in Section 2.1 and Section 2.2. Based on the isotherms, both the qualitative (ΔA) and quantitative (ΔG_mix_) parameters of the interactions were calculated. In Figure 6 and Figure 7, these are presented as molar fractions of hBest1 (X_hBest1_). The deviation ΔA (A_exp_ − A_add_) in the average experimental molecular areas A_exp_ derived from the π/A isotherms of the hBest1/POPC/Chol and hBest1/SM/Chol monolayers from the additive molecular areas (A_add_) and calculated with the additivity rule is a very important parameter revealing the intermolecular interactions between the monolayer components. The area deviations in the hBest1/POPC/Chol and hBest1/SM/Chol monolayers formed in the absence and presence of Ca ions at four different surface pressures below π_tr_ are presented in Figure 6A,B and Figure 7A,B, respectively. 

Regardless of the surface pressure and the presence of Ca^2+^, the ΔA in the hBest1/POPC/Chol monolayers have negative or zero values at X_hBest1_ < 0.02, corresponding to the biologically relevant conditions in the cell membrane and positive values at X_hBest1_ > 0.02. The ΔA values of the hBest1/SM/Chol monolayers are positive, except the one at X_hBest1_ = 0.006, which oscillates at around zero (Figure 7A,B insets). The negative values suggest that in the ternary hBest1/POPC/Chol monolayers, the attractive interactions between hBest1 and the lipid molecules are stronger than the hBest1–hBest1 and lipid–lipid interactions, thus compacting the film and promoting the miscibility. The positive ΔA values arise from the attractive interactions between hBest1–hBest1 and lipid–lipid molecules and repulsive between hBest1 and lipids, suggesting phase separation between hBest1 and lipids in the monolayers. The values of ΔA that lay on the additive line suggest a similar interaction strength between all of the molecules when referring to either the mixing or complete phase separation of the monolayer components. The higher the protein content, the higher the positive deviation from the ideal mixing and the probability for the phase separation of the components. This deviation is the most pronounced at low surface pressure. The compaction of the monolayers accompanied by an increase in the surface pressure significantly reduces the deviation in the experimental molecular areas from the additive ones, which implies better mixing of the components. The addition of Ca^2+^ ions increases ΔA, indicating enhanced separation compared to the monolayer without Ca^2+^. 

In order to analyze the π/A isotherms of these two ternary systems using Goodrich’s method [37] and to calculate the total free energy of mixing $∆G_{mix}^{\pi }$, we considered the system to be binary, in which one of the components was hBest1 and the other was the lipid mixture (POPC/Chol and SM/Chol) (see Materials and Methods). It is well known that the equimolar POPC/Chol and SM/Chol monolayers are very stable with high affinity, quite strong interactions, and a particularly favorable arrangement between the two types of lipid molecules [28], which allow us to study the effect on the inclusion of the protein hBest1 in these two-component lipid monolayers.

The negative $∆G_{mix}^{\pi }$ values of the hBest1/POPC/Chol and hBest1/SM/Chol monolayers demonstrate that the ternary monolayers are more stable than the single hBest1 and lipid monolayers; therefore, the mixing of hBest1 and lipid molecules is a spontaneous and thermodynamically favorable process that is strongly favored by increasing the molar fraction of hBest1 and the surface pressure and weakly favored by the addition of Ca^2+^ (Figure 6C,D and Figure 7C,D). In our previous studies with binary hBest1/POPC and hBest1/SM monolayers, we reported phase separation between POPC and hBest1 and spontaneous mixing between SM and hBest1 [20,29]. The results presented here show that the effect of cholesterol on miscibility/phase separation in ternary films is significant. Cholesterol enhances mixing and stability in hBest1/POPC/Chol films by reducing phase separation between hBest1 and POPC, while in hBest1/SM/Chol films, miscibility is maintained, albeit with increasing $∆G_{mix}^{\pi }$.

The miscibility or phase separation between the transmembrane protein hBest1 and lipid molecules has a direct effect on the association of the protein with lipid rafts [29], its conformation and its channel function.

## 3. Materials and Methods

All reagents and chemicals were supplied by Sigma–Aldrich (St. Louis, MO, USA) unless otherwise indicated. 

### 3.1. Cell Cultures and Purification of hBest1

MDCKII II (ATCC, CRL-2936) cells stably transfected with hBest1 were grown in DMEM with 10% fetal calf serum (FCS) and 500 μg/mL G418 salt at 37 °C and 5% CO_2_ [38,39]. hBest1 extraction and purification from MDCK-hBest1 cells were carried out as described in [39]. The method of Smith et al. (1985) was used to determine the concentration of purified hBest1 [40].

### 3.2. Monolayers Experiments

All experiments were performed under identical experimental conditions on a Langmuir balance (Kibron inc., Finland) with a Teflon trough (the total area is 194,7 cm^2^) with a Wilhelmy dynamometric system. All monolayers were assembled in a subphase containing 150 mM NaCl or in a subphase containing 150 mM NaCl supplemented with 0.5 µM CaCl_2_. The POPC/Chol and SM/Chol monolayers were formed by spreading 1 mM POPC or 1 mM SM solutions in chloroform followed by 5 min solvent evaporation and the addition of 1 mM Chol solution in chloroform (molar ratio of both lipids 1:1). After 5 min (solvent evaporation), the monolayers were compressed. To constitute the ternary hBest1/POPC/Chol and hBest1/SM/Chol monolayers, initially, 1 mM solutions of POPC or SM were spread, and then 1 mM cholesterol was added. Each addition of a different lipid was followed by 5 min for solvent evaporation. Finally, 15 µM hBest1 suspension was spread and equilibrated for 30 min before compression. For the hBest1/Chol monolayers, a molar ratio of 1:58.5 was used. In all cases, the compression and expansion rate was 40 mm/min, and the temperature of the subphase was maintained at 35 ±2 ºC. The surface compressibility moduli were obtained as numerical derivatives of π/A isotherms using the equation *C^−1^_s_ = −A_π_(∂π/∂A)_T_* [20,29]. At least three independent measurements were performed for each experimental condition.

### 3.3. Analysis of the Miscibility and Stability in Insoluble Two-Component Monolayers

A quantitative thermodynamic analysis of the miscibility and stability in the ternary monolayers containing hBest1/POPC/Chol and hBest1/SM/Chol was carried out based on the Goodrich concept [37] and as described in [20,21,29]. $∆G_{exc}^{\pi }$ can be determined by integrating the π/A isotherms of the mono-component hBest1 and the two-component POPC/Chol and SM/Chol monolayers up to the desired surface pressure:

(1) $$\Delta G_{exc}^{\pi }=N_{A}(\int_{0}^{\pi }A_{hBest1+Lipids}d\pi -X_{hBest1}\int_{0}^{\pi }A_{hBest1}d\pi -X_{Lipids}\int_{0}^{\pi }A_{Lipids}d\pi )$$

Here, A_hBest1+Lipids_ is the mean molecular area of the ternary protein–lipid (hBest1+Lipids) monolayers at given surface pressure π, where lipids hold for POPC/Chol or SM/Chol. A_hBest1_ and A_Lipids_ are the molecular areas of hBest1 and lipids in their monolayers and have the same π-value. X_hBest1_ and X_Lipids_ are the corresponding molar fractions of these components in the ternary films.

The total free energy of mixing $∆G_{mix}^{\pi }$ can be calculated as:

(2) $$\Delta G_{mix}^{\pi }=\Delta G_{exc}^{\pi }+\Delta G_{ideal}^{\pi }=\Delta G_{exc}^{\pi }+RT(X_{hBest1}lnX_{hBest1}+X_{Lipids}lnX_{Lipids})$$

Here, $∆G_{ideal}^{\pi }$ is the Gibbs free energy of ideal mixing.

## 4. Conclusions

Here, for the first time, we reported on the design and thermodynamic analysis of ternary self-assembled monolayers composed of the transmembrane Ca^2+^- dependent Cl^−^ channel bestrophin-1 (hBest1) and membrane-building lipids POPC, SM and Chol and that were designated as hBest1/POPC/Chol and hBest1/SM/Chol monolayers. In addition, we studied the effect of Ca ions on the thermodynamic state of these monolayers. 

The negative $∆G_{mix}^{\pi }$ values of the hBest1/POPC/Chol and hBest1/SM/Chol monolayers demonstrate that the ternary monolayers are more stable than the single hBest1 and lipid monolayers; therefore, the mixing of hBest1 and lipid molecules is a spontaneous and thermodynamically favorable process that is triggered by an increase in the molar fraction of hBest1 and surface pressure and less by the addition of Ca^2+^. The addition of Ca^2+^ ions was found to exert a condensing and stabilizing effect on both ternary monolayers despite their different phase states. The hBest1/POPC/Chol monolayer was in an LE state regardless of the extent of compression, while the hBest1/SM/Chol monolayer was in a more condensed state and underwent first-order LE-LC phase transition. 

Neither of the ternary monolayers analyzed here show the existence of an S state typical of Chol monolayers; therefore, the molecules of Chol are well-mixed with the other monolayer components. At biologically relevant conditions (surface area ratio protein:lipid molecules less than 1:3 and temperature of 35 ± 2 °C), the attractive interactions between the hBest1 and lipid molecules are stronger than the protein–protein and lipid–lipid interactions, thus compacting the film and promoting the miscibility. The higher the protein content, the higher the positive deviation from the ideal mixing and the probability for the phase separation of the components.

The results presented here show that the effect of cholesterol on miscibility in ternary films is significant. Cholesterol enhances mixing and stability in hBest1/POPC/Chol films by reducing the phase separation between hBest1 and POPC, while in hBest1/SM/Chol films, miscibility is maintained, even with increasing $∆G_{mix}^{\pi }$. For the hBest1/SM/Chol monolayers, the negative values of ΔG_mix_^π^ are retained and equalized with the values of ΔG_mix_^π^ in hBest1/POPC/Chol films. 

Fine-tuning the balance between the miscibility or the phase separation of the transmembrane protein hBest1 and lipid molecules has a direct consequence on its binding to lipid rafts [29], its conformational dynamics and its channel function.

## Data Availability

Not applicable.

## Sample Availability

Samples of the compounds are available from the authors.
